# The regulatory function of piRNA/PIWI complex in cancer and other human diseases: The role of DNA methylation

**DOI:** 10.7150/ijbs.68221

**Published:** 2022-05-09

**Authors:** Dong-Dong Jia, Hui Jiang, Yi-Fei Zhang, Yu Zhang, Li-Li Qian, Yin-Feng Zhang

**Affiliations:** 1 Institute for Translational Medicine, The Affiliated Hospital of Qingdao University, College of Medicine, Qingdao University, Qingdao, 266021, China.; 2 Department of Vascular Surgery, The First Affiliated Hospital, Sun Yat-sen University, Guangzhou, China.; 3 Department of Radiation Oncology, Sun Yat - Sen University Cancer Center, State Key Laboratory of Oncology in Southern China, Collaborative Innovation Center for Cancer Medicine, Guangzhou, China.

**Keywords:** piRNA, PIWI, DNA methylation, cancer, disease, diagnosis, therapy

## Abstract

Piwi-interacting RNAs (piRNAs) are a class of short chain noncoding RNAs that are constituted by 26-30 nucleotides (nt) and can couple with PIWI protein family. piRNAs were initially described in germline cells and are believed to be critical regulators of the maintenance of reproductive line. Increasing evidence has extended our perspectives on the biological significance of piRNAs and indicated that they could still affect somatic gene expression through DNA methylation, chromatin modification and transposon silencing, etc. Many studies have revealed that the dysregulation of piRNAs might contribute to diverse diseases through epigenetic changes represented by DNA methylation and chromatin modification. In this review, we summarized piRNA/PIWI protein-mediated DNA methylation regulation mechanisms and methylation changes caused by piRNA/PIWI proteins in different diseases, especially cancers. Since DNA methylation and inhibitory chromatin marks represented by histone H3 lysine 9 (H3K9) methylation frequently cooperate to silence genomic regions, we also included methylation in chromatin modification within this discussion. Furthermore, we discussed the potential clinical applications of piRNAs as a new type promising biomarkers for cancer diagnosis, as well as the significance of piRNA/PIWI protein-associated methylation changes in treatment, providing disparate insights into the potential applications of them.

## Introduction

Piwi-interacting RNAs (piRNAs) are a class of short chain noncoding RNAs, that are constituted by 26-30 nucleotides (nt) and have a 5'-terminal uridine or tenth position adenosine bias, lacking explicit secondary structure motifs 1. These short chain RNAs were firstly found in germline cells and are considered critical regulators of germline maintenance. A growing number of findings has now expanded our understanding of piRNAs biological significance, which suggests they could also somatically regulate gene expression via epigenetic alternations such as DNA methylation, transposon silencing and chromatin modification and cause diverse diseases including cancers 2-4. As a type of chemical modification, DNA methylation is the first described and well-characterized in humans and plays a significant role in long-term gene silencing, especially in the promoter regions 5, 6.

## piRNA/PIWI protein

### Structure and biosynthesis of piRNAs

**Figure 1:**
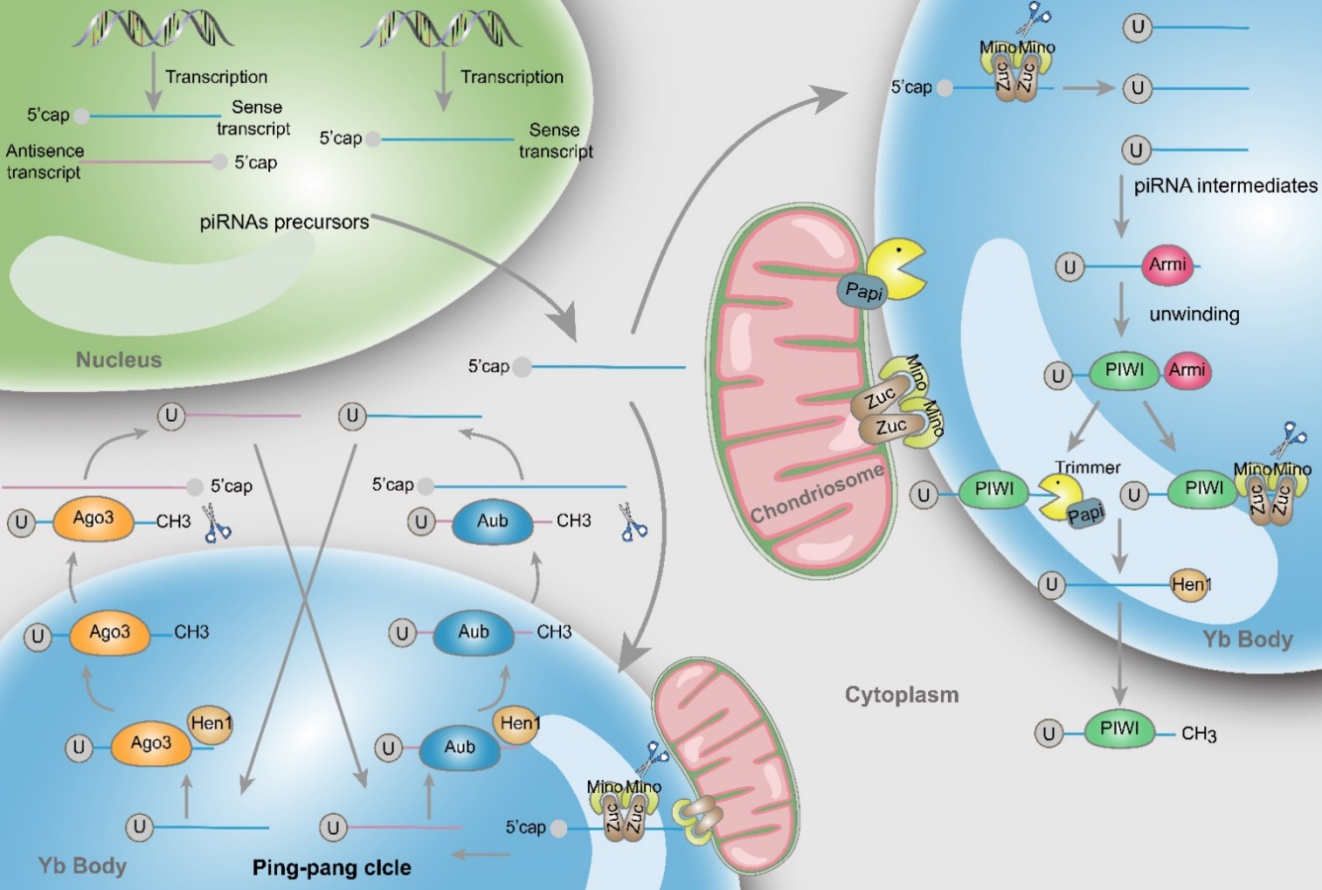
** Structure and biosynthesis of piRNAs.** Initially, two types piRNA precursors are generated from piRNA clusters within the nucleus and transcribed to the cytoplasmic Yb body to produce the primary piRNA intermediates with a 5' uracil through the incision of Zuc and its co-factors Mino. In primary amplification, after being unwound by Armi, piRNA intermediates couple with PIWI and are cleaved by Zuc or Papi-dependent trimmer to form 3'end. Following methylation of Hen1, the mature piRNA/PIWI complex is produced in cytoplasm. In secondary amplification, two type piRNA intermediates couple with Ago3 or Aub proteins to form piRNA/Ago3 or piRNA/Aub complexes, incise and generate new piRNAs. Newly generated piRNAs synthesize other piRNAs in a similar way.

Mature piRNAs are 26-30 nt in size with a 2'-O-methylation at the 3' end, which are distinct and unique features of all piRNAs 7. The difference with miRNAs and siRNAs is that piRNAs precursors are single-stranded and their secondary hairpin structures are not obvious. These precursors are generally produced from specific genomic regions that contain repeating elements, and a dicer-independent pathway typically coordinates this process. Furthermore, additional posttranscriptional modifications are required before nascent piRNAs maturing. Two major pathways are involved in the biogenesis of piRNAs: the primary and secondary amplification cycle - the latter is usually also referred to as “ping-pong cycle” 8 (Fig. 1).

#### Primary amplification

piRNAs are generated from piRNA clusters, a relatively few and characteristic genomic regions 9. Then, primary piRNAs are transported to the cytoplasm and Zucchini (Zuc) and its cofactor Minotaur (Mino) cleave them to produce piRNA intermediates with characteristic of 5' uracil 10, 11. Later, PIWI binds piRNA intermediates through the interaction of the PAZ domain and 5' uracil, and then the maturity of piRNAs is experienced the Zuc endonuclease cleavage at 3'-end, or the Papi-dependent trimmer 12. Subsequently mature piRNA/PIWI complex is yielded via methylation by Hen1 13, 14. Finally, these complexes return to the nucleus where they can activate the transcription silencing machinery and prevent transcription of their target genes.

#### Secondary amplification (Ping-pong cycle)

In the cytoplasm, after the generation of primary piRNAs, piRNAs are amplified with the ping-pong mechanism 15. This process is featured by the formation of piRNA/Ago3 or piRNA/Aub complexes, which is different from the primary synthesis associated with PIWI proteins. Specifically, Aub incises sense piRNA precursors by coupling with antisense-strand piRNAs, generating sense piRNAs that load onto Ago3. Inversely, antisense piRNA precursors are incised and generate antisense piRNAs in combination with Aub through Ago3 coupling with sense-strand piRNAs 16. Ultimately, these piRNAs combine with PIWI proteins and migrate back to the nucleus, silencing the expression of target genes. Actually, through the above mentioned amplifying mechanism, the formation of substrates for other functional piRNAs are produced accompanied by the production of piRNAs 17.

### PIWI protein

Across different species, the PIWI protein family is highly conservative 18. Four PIWI proteins are known in humans, namely PIWIL1, PIWIL2, PIWIL3 and PIWIL4, which are also respectively called HIWI, HILI, HIWI3, HIWI2. In mice, there are three PIWI homologs, PIWIL1 (also termed as MIWI), PIWIL2 (termed as MILI) and PIWIL4 (termed as MIWI2). Three PIWI proteins have also been described in Drosophila, which are named PIWI, Aub and Ago3 19-21. PIWI proteins are the members of Argonaute family, PIWI subfamily 18. As with all Argonaute proteins, PIWI proteins incorporate N, PAZ, MID, and PIWI domains, among which PAZ and MID recognize the 3′ and 5′ ends of piRNA intermediates, respectively 22, 23. The PIWI domain has endonuclease activity that allows it to incise RNA. PIWI and cancer together with other diseases currently remain an area of active research, because it has been discovered the aberrant expression of PIWI proteins is closely related to adverse clinical outcomes in cancerous patients 24. In addition, several studies have also revealed that PIWI proteins have a strong correlation with DNA methylation in cancers. For instance, MILI and MIWI2 were initially found to play a crucial part in the occurrence of de novo DNA methylation of retrotransposons 25. Further studies indicated that MILI and MIWI2 have differential functions on DNA methylation. MILI is responsible for the DNA methylation of a wider range of TEs while MIWI2 is implicated in DNA methylation and transcriptional gene silencing 26, 27.

### Function of piRNAs

**Figure 2:**
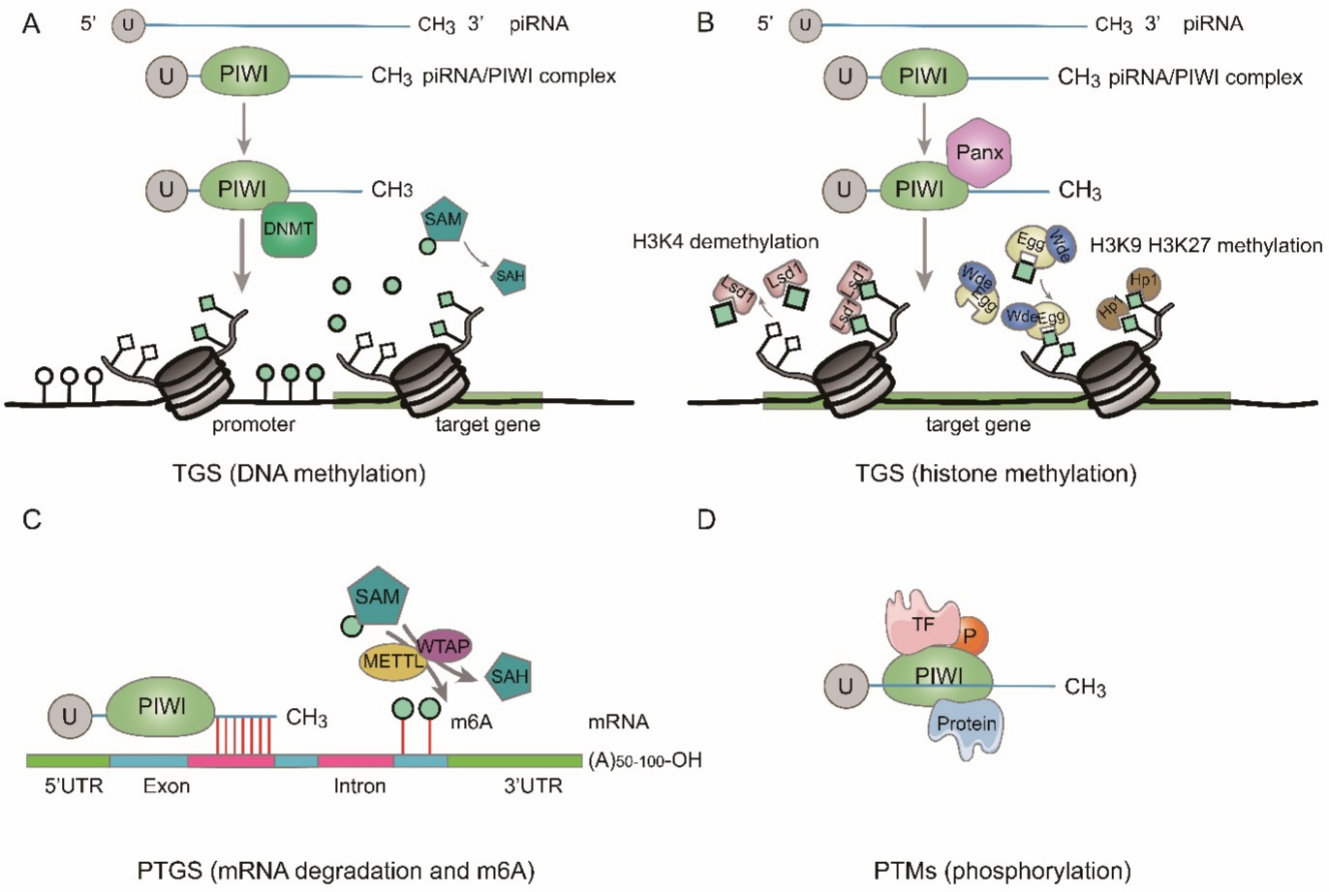
** Function of piRNAs. A.** DNA methylation. At transcriptional gene silencing (TGS) level, the piRNA/PIWI complex methylates CpG sites of target gene by recruiting DNMT. **B.** Histone methylation. At TGS level, the piRNA/PIWI complex recruits repressive H3K9me3 and H3K27me3 marks to target gene and removes active H3K4me2 marks from promoter region. **C.** mRNA degradation and m6A methylation. At post-transcriptional gene silencing (PTGS) level, piRNA/PIWI complex inhibits the function of targeted RNAs by sequence complementary or block the m6A methylation of mRNA transcripts by interacting with methytransferase-like proteins (METTL) and/or Wilms' tumor 1-associating protein (WTAP). **D.** The piRNA/PIWI complex binds to transcriptional factor (TF) to regulate posttranslational modifications (PTMs).

According to previous studies, piRNAs function at the transcriptional, posttranscriptional and posttranslational modifications levels 28-31 (Fig. 2).

#### Transcriptional gene silencing (TGS)

By binding to the coding regions of gene promoters, piRNAs are involved in the gene regulation process 32. It was discovered that the piRNA/PIWI pathway is implicated in the transcriptional gene silencing (TGS) through DNA methylation and histone modifications 20, 33. When formed in the cytoplasm, the piRNA/PIWI complex comes into the nucleus and couples with the genomic target to form a fresh complex. After the new complex combining with Panoramix (Panx), it recruits silencing constituents, and TGS begins. Firstly, activated histone 3 lysine 4 dimethylation (H3K4me2) markers are removed from promoter regions by lysine-specific demethylase 1 (Lsd1), thus the transcription of RNA Pol II is inhibited 34. Then, repressive histone 3 lysine 9 trimethylation (H3K9me3) markers are added to the target DNA by Eggless (Egg) and its cofactor Windei (Wde). Subsequently, heterochromatin protein 1 (Hp1) is recruited, leading to heterochromatin formation (Fig. 2b). Besides, the piRNA/PIWI complex also methylates genic CpG sites *(nontransposon loci)* by recruiting DNA methyltransferase (DNMT) and alters transcriptional activity 25 (Fig. 2a).

#### Posttranscriptional gene silencing (PTGS)

After discovering TGS, researchers found that piRNAs could suppress the function of target genes by regulating posttranscriptional networks, which is similar to what miRNAs form, namely, piRNA-RNA interactions. lncRNAs 35, mRNAs 36, etc. are included in RNAs that can interact with piRNAs. Base pairing at the 5'-end of piRNAs are required in the process of piRNA interaction, with 2-11 nt for strict base pairing and 12-21 nt for less stringent base pairing 37 (Fig. 2c). Moreover, by recruiting CCR4 NOT (carbon catabolite-repressed 4-negative on TATA-less) and Smaug, piRNA/PIWI complex can facilitate RNA repression to construct a specific piRNA-induced silencing complex (pi-RISC), and the latter can cause incomplete base-pairing with RNA 38. For example, Peng L, et al. discovered that piR-55490 could bind to the 3'-UTR of mammalian target of rapamycin (mTOR) resulting in mRNA degradation and inhibiting the occurrence and development of lung cancer (LC) 36. Additionally, several studies revealed that piRNA could function in the process of posttranscriptional gene regulation by another way, such as m6A RNA methylation 39, 40 (Fig. 2c). For example, Gao et al. demonstrated that a cardiac-hypertrophy-associated piRNA (CHAPIR) combined with PIWIL4 to form CHAPIR-PIWIL4 complex. Afterward, the CHAPIR-PIWIL4 complex interacted directly with METTL3 and blocked Parp10 mRNA transcripts m6A methylation, resulting in upregulation of Parp10 expression and ultimately leading to the progression of pathological hypertrophy and cardiac remodeling 39. Additionally, piRNA-30473 was involved in tumorigenesis of diffuse large B-cell lymphoma by manipulating m6A RNA methylation 40.

#### Posttranslational modifications (PTMs)

The piRNA/PIWI complex can directly interact with certain transcriptional factors (TFs) and regulates their posttranslational modifications such as phosphorylation (Fig. 2d). For example, in colorectal cancer (CRC), researchers found that piR-823 increased the activity of heat shock factor 1 (HSF1) by binding to it and facilitated Ser326 phosphorylation, leading to over-expression of heat shock proteins and proliferation of CRC cells. Moreover, piR-54265/PIWIL2 complex interacted with signal transducer and activator of transcription 3 (STAT3) and phosphorylated-SRC (p-SRC) to form complex and activated STAT3 phosphorylation, in which both of them promoted CRC tumorigenesis 41, 42.

### piRNA/PIWI complex affects DNA methylation

Based on the existing research evidence, at the transcriptional level, piRNAs undergo epigenetic changes mainly via sequence-specific aberrant DNA methylation and histone modification in somatic cells 43. The crosstalk between piRNAs and DNA methylation has profound implications for genomic stability and gene expression, which may lead to aberrations in cell signaling transduction pathways, ultimately disease occurrence 44, 45. DNA methylation plays an important role in epigenetic mechanisms by silencing transposons and other repetitive elements, and stably repressing specific transgenes and endogenous genes 46. The methylation site is mostly the 5'-cytosine-phosphate-guanine-3' (CpG) cytosine dinucleotide, and rarely a non-CpG sequence 47. CpG dinucleotides are localized and clustered to form CpG islands, detected at the majority of mammalian promoter regions 48. In the human genome, the methylation status of 28 million CpG dinucleotides determines the gene expression level through the grading status of genes and regulatory sections 49. Four types of DNA methyltransferases (DNMTs) are responsible for DNA methylation and methylation patterns: DNMT1, DNMT3A, DNMT3B and DNMT3L 50. DNMT3A and DNMT3B contribute to de novo DNA methylation and DNMT3L facilitate the methylation of retrotransposons by interacting with DNMT3A and 3B, whereas DNMT1 recruits methyl groups to hemi-methylated CpG sites to maintain cytosine methylation modification in the new synthetic chain 51.

#### DNA methylation at transposon loci: the piRNA/PIWI complex mediates TGS by causing transposon DNA methylation

Ago family proteins assemble with small RNAs into RISC, such as miRNAs, siRNAs or piRNAs, mediating sequence-specific target gene silencing 52. RISC assembly of piRNAs begins by loading single-stranded piRNA intermediates into PIWI subfamily proteins and directing the PIWI proteins to transposon element (TE) targets 52, 53. Depending on the replication mode, TEs can be divided into two types: 1) retrotransposons, transcribed into RNA intermediates, and 2) DNA transposons, mobilized without transcription 54.

TEs are thought to be kept silent by DNA methylation and packaging into heterochromatin 55. Previous evidence indicated that piRNAs, together with their PIWI proteins, could promote de novo DNA methylation of retrotransposons by generating complexes, thereby inhibiting transposons and maintaining gene stability 27, 28. The loss of DNA methylation may stimulate gene expression at TEs including LINE-1 56. Notably, by integrating into the genome, active retrotransposons can cause genomic variations, change chromatin structures and alter the proximal gene expression 57. Uncontrolled transposons threaten the integrity of the genome, and these mutations can be passed on to the next generation 58, 59. Indeed, activation of LINE-1 may be involved in carcinogenesis and a large proportion of cancer patients have somatic retrotranspositions of LINE-1 elements 56. In addition to DNA methylation, activated TEs can also be epigenetically silenced through the way of piRNA-dependent heterochromatin formation 60. Consequently, the piRNA/PIWI complex plays crucial roles in maintaining minimum levels of transposons and protecting genomic stability 61.

### DNA methylation at nontransposon loci: piRNA increased the expression of DNMTs and silenced tumor suppressor genes by methylation

Moreover, piRNAs can induce and affect DNA methylation not only at transposons, but also at nontransposon loci 29. When transfecting piRNA mimics into human somatic cells, methylation changes at multiple genome sites were found, signifying that piRNAs are essential not only for methylation of transposons but also specific genes. According to epigenetic analysis of piRNAs in gene-specific DNA methylation, it is suggested that single-copy piRNAs contribute to DNA methylation at specific genome loci by incompletely combination with genomic DNA at non-TE sites 29. For example, it was reported that piRNA-823 recruited the DNMT3A and DNMT3B, increased tumor suppressor p16INK4A DNA methylation and inhibited its expression in multiple myeloma cells 44.

## The piRNA/PIWI complex affects the occurrence and development of diverse diseases through DNA methylation

**Figure 3:**
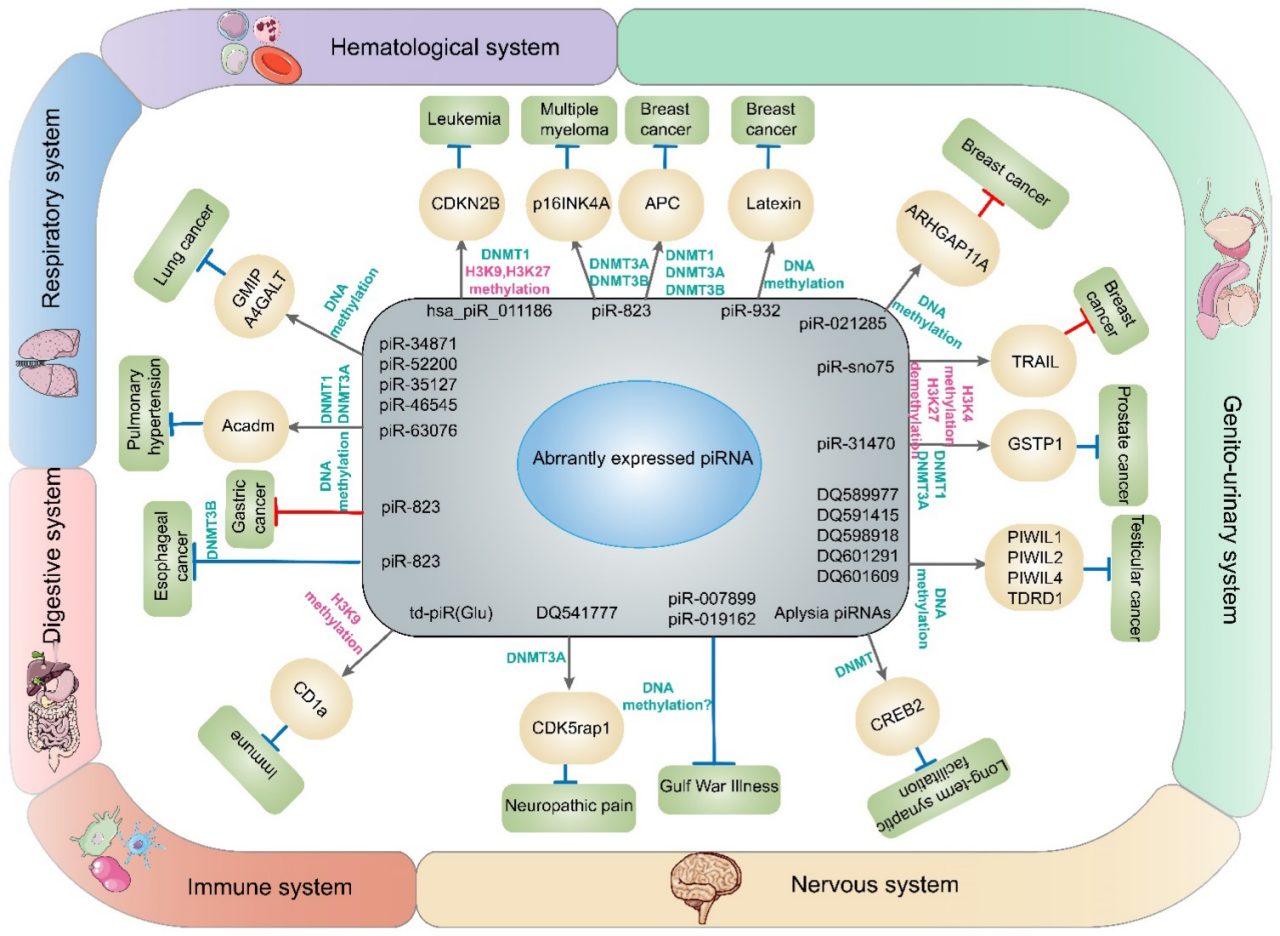
** piRNAs and DNA methylation involved in diseases in this article.** The dysregulation of piRNAs contributes to abnormal expression of genes through DNA methylation or histone methylation and finally promotes or restrains the development of cancers or other diseases in different systems.

**Figure 4:**
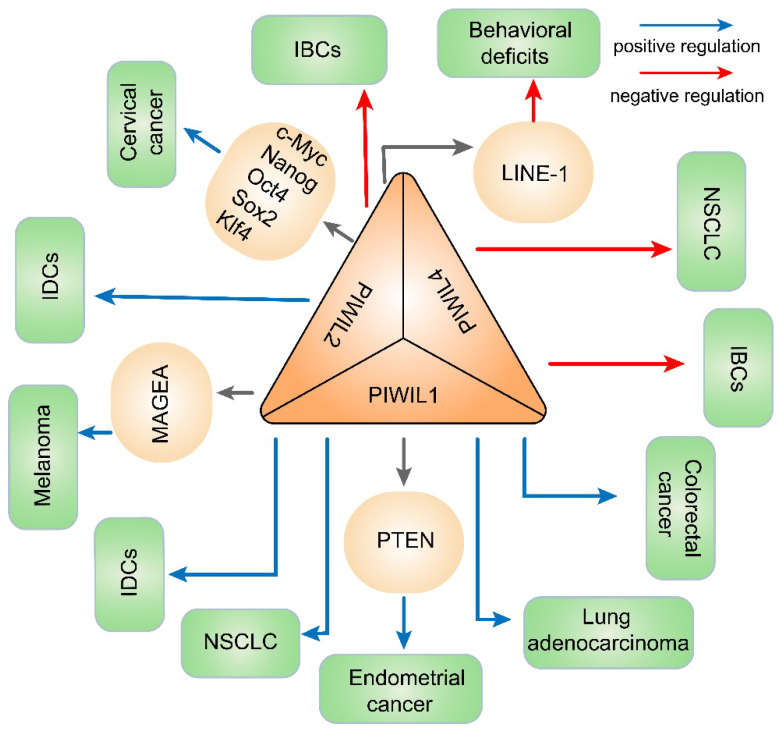
** PIWI and DNA methylation involved in diseases in this article.** The dysregulation of PIWI protein contributes to abnormal expression of genes through DNA methylation and finally promotes or restrains the development of diseases.

Recently, second-generation sequencing technology has provided an extremely effective way to research disease-related genes and noncoding RNAs including piRNAs 62. Since piRNAs were found to be involved in gene regulation, there has been increasing interest in what roles they play in human diseases. Many studies have shown that the dysregulation of piRNAs could facilitate or inhibit the occurrence and development of multiple diseases, especially cancer. Epigenetic alterations in cancer comprise global DNA hypomethylation, gene-specific DNA hypermethylation, histone modification, etc., causing activation of oncogenes such as Ras and cyclin D2 63, 64 and silencing antioncogenes such as Rb1 and p16 65. Global DNA hypomethylation may be related to genomic instability; for instance, demethylated transposons can damage the genome when transcribed or transposed to other genomic regions 66. Nonetheless, gene-specific hypermethylation tends to occur in the promoter regions of antioncogenes which may induce or promote cancer by silencing genes involved in DNA repair, apoptosis and tumor-specific signaling pathways 67. Previous studies have discovered that ncRNAs, including miRNA, circRNA and lncRNA, could regulate gene expression through DNMT mediated DNA methylation 68-70, but the role of piRNAs in the DNA methylation of diseases such as cancer has not been systematically described. Here we summarized the regulation and function of piRNAs/PIWI proteins in DNA methylation in cancers and other human diseases (Tables 1 & 2; Figures 3 & 4). At the same time, we also mentioned chromatin methylation modification, which plays a significant role in gene expression regulation along with DNA methylation.

### The piRNA /PIWI complex influences various cancers by regulating DNA methylation

#### Breast cancer

In women, breast cancer (BC) is the most commonly diagnosed cancer and the leading cause of cancer-related death 71. Currently, BC affects more than 10% of women worldwide, especially those before the age of 45 72, 73. BC consists of mainly four molecular subtypes, namely basal, luminal A, luminal B, HER2-overexpressing, and they have diverse clinical courses, prognoses and therapies 74, 75. Nevertheless, their underlying molecular mechanisms remain poorly understood. Despite medical advances in early screening and diagnosis, BC mortality rate has continued to rise worldwide over the past 25 years, which could be attributed to the increased incidence and prevalence of this cancer 76. Therefore, finding new strategies and approaches to prevent BC, improve survival rates, and reduce cancer mortality is crucial and urgent. Recent studies have found that piRNAs could influence BC tumorigenesis, growth, invasiveness, and metastasis by methylating a specific gene site, implying that piRNAs could be used as a class of biomarkers for the early diagnosis of BC and as an alternative treatment 77-80.

First, it was revealed that the changes in DNA methylation caused by piRNAs in BC could promote tumor development. For example, piR-823 was reported to increase the expression of DNMTs including DNMT1, DNMT3A, and DNMT3B, and promote DNA methylation level of the adenomatous polyposis coli (APC) gene, a tumor suppressor, thereby motivating WNT signaling pathway and inducing the formation of cancer cell stemness (CSCs) in the luminal subtype of BC cells, ultimately contributing to BC tumorigenesis 77. Similarly, piR-932 was also proven to induce BC metastasis through gene-specific hypermethylation 78. piR-932 expression was significantly higher in BC cells that were induced by EMT, and it could form complexes with PIWIL2. Then, the piR-932/PIWIL2 complex reduced Latexin expression through improving the methylation of CpG island in promoter region. Latexin, also a tumor suppressor, could reduce the transformation of old stem cell into CSCs, decrease the cell replication, and enhance the apoptosis 81, 82. In contrast, there are some piRNAs that can inhibit BC growth through methylation 79, 80. The variant piR-021285-mimic transfected into BC cell lines could weaken methylation of pro-invasive gene ARHGAP11A at the region of the 5' UTR/first exon CpG island, leading to increased ARHGAP11A expression and tumor cell invasiveness 79. Conversely, wild type piR-021285 was proven to inhibit BC cell proliferation and invasion by improving ARHGAP11A methylation. Similarly, a GAS5-derived small RNA (pi-sno75) also played an inhibitory role in tumor growth by inducing methylation/demethylation 80. Interestingly, pi-sno75 constituted the piRNA/PIWI complex by binding to the PIWIL1/4 proteins and affected gene expression through a different mechanism. The piRNA/PIWI complex coupled with WDR5 and subsequently recruited the COMPASS-like complex containing MLL3/UTX to the promoter region of tumor necrosis factor (TNF)-related apoptosis-inducing ligand (TRAIL). TRAIL, a proapoptotic protein, was significantly upregulated by increasing H3K4 methylation/H3K27 demethylation, consequently leading to the inhibition of tumor growth 80.

In addition to piRNAs, PIWI was also significantly associated with DNA methylation in BC 83. First, epigenetic inactivation of the PIWIL2 gene was caused by hypermethylation of the promoter CpG island and delocalization of the PIWI protein into cytoplasmic stress bodies 84. Furthermore, DNMT1, histone H1, HP1 and SUV39H1 were downregulated in invasive breast cancers (IBCs) with PIWIL2 underexpression, while DNMT1 was downregulated only in IBCs with PIWIL4 underexpression. Later, molecules involved in genome methylation, chromatin accessibility and increased inflammatory/cytotoxic immune reactions were potentially reduced, and IBCs were thereby promoted 83. In addition, research indicated that PIWIL1 and PIWIL2 expression were significantly increased in invasive ductal carcinomas (IDCs), inducing a stem-like state of cancer cells through abnormal DNA methylation, generating genomic silencing and ultimately promoting cancer development 85.

### Lung cancer

Lung cancer (LC) has the highest cancer-related mortality among men and the second-highest cancer-related mortality (after breast cancer) among women worldwide 86. Non-small cell lung cancer (NSCLC) is a predominant type of LC, accounting for 85% of all lung cancers, while the incidence of small cell lung cancer is low, accounting for only 15% of all lung cancers 87. Patients with LC have a poor prognosis and are usually diagnosed late in the disease, leading to a high mortality rate 88. Epigenetic alterations in LC, including DNA methylation, play critical roles in cancer progression, and epigenetic recognition of LC pathogenesis is increasingly playing a significant role in the discovery of novel diagnostic biomarkers and the development of therapeutic strategies 89.

Firstly, studies have shown that the DLK1-DIO3 locus is methylated aberrantly in current and former smoker patients with NSCLC and aberrant expression of DLK1-DIO3 might contribute to tumorigenesis in LC 90. Previous studies indicated that the DLK1-DIO3 locus encodes piRNAs in somatic cells and might enhance the prognostic potential of piRNAs to specifically predict patient outcome. In addition, they could have great potential as novel biomarkers and therapeutic targets 91. However, how abnormal methylation of the DLK1-DIO3 locus affects LC through piRNAs remains to be further studied. In addition, researchers have recently demonstrated a new RASSF1C-PIWIL1-piRNA pathway that facilitates LC cells progression and migration by regulating the methylation of oncogenes and anti-oncogenes 92. In LC cells, Ras Association Domain Family Member 1 isoform C (RASSF1C) enhanced the PIWIL1 gene expression by the MEK-ERK1/2 pathway 93. Afterward, PIWIL1 and its interacting piRNAs (piR-34871, piR-52200, piR-35127 and piR-46545) regulated DNA methylation of oncogenes and tumor suppressor genes and inhibited cell apoptosis 2, 94. In this way, the Gem Interacting Protein (GMIP) gene, which has anticancer properties, was hypermethylated and downregulated. Conversely, the A4GALT gene which serves as an oncogene was upregulated by demethylation. Consequently, cancer cells would initiate local tumor growth at the site of metastasis by transforming from mesenchymal cells to epithelial cells 92. In terms of PIWI protein, PIWIL1 expressed aberrantly in lung adenocarcinoma and NSCLC due to promoter DNA hypo-methylation and promotes proliferation, invasion and migration of cancer cells 95. For another PIWI protein, underexpression of PIWIL4 was positively correlated with overall methylation in NSCLC 96. Moreover, in tumor tissues, PIWIL4 expression was downregulated and patients with lower PIWIL4 expression had a shorter time to relapse (TTR) and overall survival (OS) than others 96.

### Hematological neoplasm

Hematological malignancies include leukemia, multiple myeloma (MM) and lymphoma 97. MM is a B cell-derived malignancy featured with the clonal replication of plasma cells in the bone marrow. Although remarkable progress has been made in treating the disease, drug-resistant relapse usually occurs and MM remains largely incurable 98. Diffuse large B-cell lymphoma (DLBCL), the most common subtype of malignant lymphoma, is heterogeneous in its clinical presentation, morphology and biological behavior 99. Although standard therapy cures two-thirds of patients, approximately 30~40% are still not cured 100. Therefore, further studies on molecular carcinogenesis mechanisms are necessary to provide new therapeutic strategies and improve prognosis.

In MM, CSCs and myeloid-derived suppressor cells (MDSCs) are two important cellular components in the tumor microenvironment, that may change the cancer phenotype and affect patient survival 101. It was reported that piR-823 expression was increased by granulocytic-MDSCs (G-MDSCs), which in turn promoted DNA methylation and increased the tumorigenic potential of MM cells 102. In contrast, silencing piR-823 in MM cells resulted in markable decrease in DNMT3A and DNMT 3B at both the mRNA and protein expression levels, which reversely resulted in a reduction in overall DNA methylation and re-expression of the methylation-silenced cancer suppressor gene p16INK4A 44, 102. Similarly, in the leukemia cell line U937, the CDKN2B-related has_piR_011186 constituted a complex with DNMT1, SUV39H1 and EZH2 proteins, upregulating the methylation level of DNA and histone H3 (including histone H3K9 and H3K27) in the CDKN2B promoter site. Thus, the expression of the CDKN2B gene was inhibited, cell cycle progression was promoted and apoptosis was reduced 103.

### Digestive system neoplasm

Digestive malignancies are a group of cancers that occur in the gastrointestinal tract and its related organs. Cancers of the colon and rectum, stomach, liver, esophagus, and pancreas are five of the ten most commonly diagnosed cancers and causes of cancer-related death 104. Among these digestive cancers, esophageal cancer (EC) is a highly aggressive malignancy that is usually detected in locally advanced stages with distant metastases, limited treatment options, and a five-year survival rate of less than 20% 105. Colorectal cancer is one of the most commonly diagnosed human cancers and the third reason of cancer-related death 106. Consequently, identifying fresh and robust diagnostic and therapeutic targets that can facilitate early detection of digestive malignancies or as a specific treatment to reduce mortality remains a priority in cancer research.

Recently, a study revealed that piR-823 induced aberrant DNA methylation through the epigenetic pathway DNMT3B and exerted an oncogenic role in esophageal squamous cell carcinoma (ESCC). Additionally, the expression of piR-823 has high specificity in ESCC, and a higher level of piR-823 signified a higher risk of lymph node metastasis, suggesting its potential as a biomarker for diagnosis and prognosis 45. Intriguingly, over the past few years, PIWI have also emerged as promising biomarkers for CRC diagnosis and prognosis 107. The ectopic expression of PIWIL1 has recently been implicated in tumor differentiation and depth of invasion, as well as lymph node invasion and metastasis 108. In addition, in a significant fraction of CRC, the PIWIL1 gene was ectopically activated accompanied by demethylation of gene promoter, together with production of germline factors required for piRNA, which is a promising application of cancer-specific immunotherapies 109. For gastric cancer, although studies have shown that piR-823 demonstrated tumor suppressor in gastric cancer cells *in vitro* and *in vivo* 110, the roles of piRNA and its associated DNA methylation in the tumorigenesis and development of gastric cancer warrant further study. However, several literatures have revealed that piR-823 inhibits cancer cells proliferation and causes an abnormal “stem-like” state in cells by reducing tumor supporter gene methylation 111.

### Genitourinary neoplasms

Genitourinary cancers typically refer to neoplasms of the kidneys, bladder, prostate, testicular, uterus, ovary and so on. Prostate cancer is the most common tumor in men worldwide 112. Current statistics show that the diagnosis rate and mortality of prostate cancer are as high as 14.3% and 2.6% respectively, or even higher. 113. In males between 14 and 44 years of age, testicular cancer is the most common solid malignancy and its incidence has risen worldwide during the past two decades 114. Although the piRNA/PIWI pathway plays a critical role in male germline development 115, 116, the role of piRNAs in genitourinary tumors is scarce and deserves further attention.

It was demonstrated that piRNAs were associated with prostate cancer via specific hypermethylation, which led to their transcriptional inactivation in prostate cancer. First, piR-31470 could form a piR-31470/PIWIL4 complex by binding to PIWIL4. Afterward, this complex coupled with the glutathione S-transferase pi 1 (GSTP1) RNA transcripts and recruited DNMT 1 and DNMT 3A and methylated the CpG island of GSTP1 to activate and sustain the hypermethylation and inactivation of GSTP1. In the process of prostate carcinogenesis, silence of GSTP1 via hypermethylation is an early and common event and functional inactivation of GSTP1 enhanced the susceptibility to oxidative stress and the risk of prostatic cancer progression 117. Otherwise, under the circumstance of downregulation of piRNA and low DNA methylation of the LINE-1 TEs, it has been observed that the repression of PIWIL1, PIWIL2, PIWIL4, and Tudor domain-containing protein 1 (TDRD1) was associated with hypermethylation of the promoter CpG island in testicular tumors 118.

Regarding to the function of PIWI protein and DNA methylation in genitourinary neoplasms, there are two main types of tumors that are common in women: cervical cancer and endometrial cancer. First, HPV oncoproteins E6 and E7 have been reported to reactivate PIWIL2 during cervical cancer tumorigenesis. Then PIWIL2 induced H3K9 acetylation but reduced H3K9 trimethylation, resulting in epigenetic reprogramming and the maintenance of embryonic stem cells (ESCs) by affecting the expression of genes in c-Myc, Nanog, Oct4, Sox2 and Klf4 119. In another female tumor, endometrial cancer, PIWIL1 caused the silence of PTEN expression through DNMT1-mediated hypermethylation 120. PTEN is a tumor suppressor with growth and survival regulatory functions 121. Therefore, the PIWIL1/DNMT1/PTEN signaling pathway may play a significant role in the progression of type I endometrial cancer 120.

### Melanoma

Melanoma is still a potentially lethal skin malignancy and the occurrence of malignant melanoma has continued to increase for many years 122. Although most patients have localized disease when diagnosed and are treated by surgical excision of the primary tumor, many patients develop metastases 123. Nevertheless, piRNA/PIWI protein might become a potential target in melanoma therapy.

MILI, also named PIWIL2 in humans, inhibited melanoma cell metastasis, probably providing a novel perspective on melanoma treatment. Specifically, MILI could methylate the LINE-1 repetitive sequence to regulate MAGEA expression and restrain melanoma cell migration 44. In addition, it has been demonstrated that aberrant MAGEA expression has a very strong correlation with high-grade lesions and terminal cancer 124.

## The piRNA/PIWI complex influences other human diseases through regulating DNA methylation

### Reproductive System Diseases

Infertility remains a global problem what is estimated to affect almost 186 million people worldwide, and human infertility contributes to more than 50% of all cases of global childlessness 125. Infertility patients bear enormous psychological and social pressure, and infertility leads to low fertility and population aging, which increases the social burden 106. piRNAs were originally discovered in mouse testis cells and further studies confirmed that they play a key role in germ cell generation and maintenance in Drosophila and mouse models. Since piRNAs act as general effectors of gene expression and epigenetic alterations during spermatogenesis, they may be useful and effective tools for diagnosing infertility. In addition, piRNA/PIWI protein can affect spermatogenesis by regulating DNA methylation and thus could also be a promising target of novel drugs for treatment.

Transposons pose a serious challenge to the germline, and mechanisms that inhibit their activity are crucial for transgenerational genomic integrity; thereinto LINE-1 is the most important retrotransposon and is epigenetically repressed by CpG island DNA methylation 126. There are three important epigenetic mechanisms that inhibit LINE-1 transposons and enhance genomic stability in spermatogenesis 25, 126, 127. Initially researchers discovered that two PIWI proteins MILI and MIWI2 played critical roles in the construction de novo DNA methylation of retrotransposons containing LINE-1 and intracisternal A particles (IAPs) in germ cells 25. Further study indicated that MIWI2 as a regulator was indispensable in germ cell's DNA methylation and gene silence 27. Later five piRNAs in spermatogenic failure samples, DQ589977, DQ591415, DQ598918, DQ601291, and DQ601609 were found to be downregulated and they targeted PIWI2 and TDRD1 gene promoters. In addition, their expression had inverse correlation with the methylation level of PIWI2 and TDRD1 gene promoters 127. Likewise, a recent study also showed that DNA methylation impacted the piRNA/PIWI complex pathway and was involved in the production of pachytene piRNA and gene expression during the bovine hybrid male sterile spermatogenesis process 128. DNA hypermethylation and disrupted piRNA production resulted in failure of germ cell development which might drive male sterility in cattle crosses. In addition to these two mechanisms, the euchromatic repressive H3K9 modification co-repressed LINE-1 expression 126. Subsequent research found that during the DNA hypomethylation stage, protein arginine methyltransferase 5 (PRMT5), together with ubiquitin like with plant homeodomain and ring finger domains 1 (UHRF1), was necessary for the repressive H2A/H4R3me2s chromatin modification on LINE-1 and IAP transposons in primordial germ cells (PGCs) and participated in transposon silencing, while conditional loss of these proteins in early PGCs caused complete male and female sterility 129, 130. Thereafter, PRMT5 translocated back to the cytoplasm and participated in the piRNA pathway, which promoted transposon silencing through de-novo DNA remethylation 129.

### Nervous System Diseases

Many publications have revealed that piRNAs exist extensively in the central and peripheral nervous systems and participate in regulating the physiological and pathological processes of various neurological diseases including anxiety disorders and Parkinson's disease 131. The study of the role of piRNAs and DNA methylation in neurological diseases may help to further raise our cognitive level of the pathogenesis of neurological diseases and develop novel diagnostic markers and therapeutic targets for these diseases.

Researchers demonstrated for the first time that Aplysia piRNA/PIWI complex facilitated serotonin-dependent methylation at the promoter CpG island of CREB2, a major inhibitory limitation of memory in Aplysia, enhancing long-term synaptic facilitation 132. Later, a growing body of research showed has shown that piRNAs have epigenetic roles in the mammalian nerve and brain that we have not explored before 133, 134. The piRNA pathway might play a role in locomotory, exploratory and normal anxiety behavior. It has been demonstrated that intergenic and LINE-1 promoter regions of brain genomic DNA was preferentially hypomethylated across the whole genome of MILI/piRNA-deficient mice, and ultimately, MILI mutant mice presented behavioral deficits such as hyperactivity and decreased anxiety 133. Furthermore, piR-DQ541777 increased the methylation degree of CpG islands by recruiting DNMT3A to the CDK5rap1 promoter and consequently reduced the expression of CDK5rap1 and regulated neuropathic pain 134. Otherwise, in Gulf War Illness (GWI), a chronic disorder distinguished by memory dysfunction and depression, changes in methylation throughout the brain and in miRNA and piRNA (piR-007899, piR-019162) expression were detected 135. However, their connection remains to be further elucidated. Another study indicated that PIWIL1 regulated polarization and radial migration of neurons in part by modulating the expression level of microtubule-associated proteins (MAPs) and mutations of PIWI might be associated with developmental cerebral disorders like autism. However, some literatures pointed out that PIWI might not regulate MAP expression through methylating target gene promoters in cortical neurons 136.

### Cardiovascular diseases

Cardiovascular diseases (CVDs) are the main causes of human mortality worldwide 137. With the advancement of next-generation sequencing technology, piRNAs are differentially expressed in CVDs, indicating that they are involved in the occurrence and progression of cardiac and vascular diseases 138.

Recent research has indicated that the piRNA/PIWI complex might be a new class of molecules that are potentially useful as biomarkers for the classification of pulmonary hypertension as well as therapeutic targets. The expression of piR-63076 was upregulated in pulmonary vessels under hypoxia and partly attributed to DNA methylation of the acyl-CoA dehydrogenase (Acadm) promoter region. Acadm deficiency ultimately increased hypoxia-induced pulmonary-artery smooth muscle cells (PASMCs) proliferation 139.

### Others

Although little is known about how piRNAs function in the human immune system, studies have suggested that piRNAs could act as signal transduction mediators in well differentiated immune cells 140.

First, a previous study showed that piR-30840 interacted with PIWIL4 and Ago4 that targeted the intron of IL-4, which inversely caused the degradation of IL-4 pre-mRNA 141. Subsequently, the reaction restrained the production of IL-4 in CD4 lymphocytes and the differentiation of Th2 CD4-T lymphocytes. The above aberrant expression has been reported in pathological conditions of certain diseases, such as asthma. This finding possibly provides a novel therapeutic target for human allergic diseases. Later, subsequent research indicated that a tRNA-Glu-derived piRNA [td-piR (Glu)] combined with PIWIL4 to form the td-piR (Glu)/PIWIL4 complex and subsequently recruited SETDB1, SUV39H1, and Hp1b to the CD1a promoter region, promoting H3K9 methylation. As a consequence, the transcription of CD1a was notably suppressed. This mechanism may provide a viable and specific method for controlling CD1a expression in the treatment of certain autoimmune diseases, and at the same time, it can also avoid transplant immune rejection and tumor immune evasion caused by abnormal expression of CD1a. Interestingly, IL-4 significantly reduced the production of tRNA-Glu, thereby reducing the generation of td-piR (Glu). However, in another immune disease, rheumatoid arthritis, the piRNA/PIWI system might not play such a significant role 142. In rheumatoid arthritis synovial fibroblasts (RASFs) and osteoarthritis synovial fibroblasts (OASFs), PIWIL2/4 mRNA expression was potently increased via TNFα+IL1β/TLR- ligands and piR-16659 was increased 4-fold upon poly (I:C) stimulation. However, silencing PIWIL2/4 affected neither LINE-1 methylation nor RASF proliferation. Instead, another system that controls LINE-1 expression, TREX-1, has been detected to be down-regulated in RASF 142.

## piRNAs and piRNA-associated methylation as potential biomarkers for cancers

In terms of mortality, globally, cancer is the second cause leading to death (8.97 million deaths) after ischemic heart disease but is expected to become the most common cause of death in 2060. Several cancers, such as esophageal, liver, and pancreatic cancers, have the worst prognosis, usually less than 20% in 5 years 86. Meanwhile, certain chronic diseases such as heart failure can force a tremendous clinical burden, disrupt social standards, and erode a huge number of economic resources 143. Accordingly, early diagnosis coupled with effective and accurate treatment is crucial.

More and more evidence point to the fact that piRNAs could represent attractive diagnostic and prognostic biomarker candidates, as some piRNAs have been involved in the development of many diseases 138, 144. First, piRNAs can be used to distinguish cancer patients from healthy individuals based on the expressive discrepancy in cancer samples and normal controls and the detection sensitivity is higher than CEA and CA19-9 107, 145. Similarly, a previous study on piRNAs in gastric cancer indicated that piRNAs provided higher sensitivity and specificity than existing biomarker detection systems based on miRNAs 145. Second, differences in piRNA expression are useful for predicting the prognosis of TTR and OS in cancer patients. piRNAs in peripheral blood could be used as disease progression indicators and postoperative long-term monitoring due to their remarkable distinct expression among preoperative and postoperative patients 145. Third, due to the protection of PIWI proteins, circulating piRNAs in peripheral blood or extracellular vesicles are highly stable and abundant, which makes them ideal biomarkers for liquid biopsy 145, 146. As an example, Lipps et al. was the first to illuminate the function of piRNAs in chronic thromboembolic pulmonary hypertension, demonstrating that piRNAs in the extracellular vesicles can be applied as biomarkers to reflect disease severity 147. In addition, in recent years, piRNA-associated DNA methylation was detected in the early stages of tumorigenesis and cancer progression and has been proposed as a biomarker for cancer detection, prognosis and prediction of treatment response 148. For example, in related breast cancer studies, it was found that early breast cancer diagnosis and the prediction of breast cancer risk could be achieved by detecting aberrant methylation of several genes in serum DNA and genome-wide epigenetic changes. Similarly, numerous studies related to colorectal cancers have been conducted to identify specific methylation markers that are important for CRC detection. In reality, clinical assays evaluating the SEPT19 gene and vimentin methylation state became commercially available 148.

## piRNA/PIWI complex-associated methylation as a potential therapeutic strategy for cancers

Beyond the role of piRNAs and piRNA-associated methylation as biomarkers, several studies have put forward their potential role as therapeutic tools 44, 78, 109, 149 just like other non-coding RNAs 150-152. On the basis of the functional characteristics of piRNAs, we offer some thoughts on piRNA as a possible target for cancer therapy as follows. Firstly, at the pre-transcriptional level, specific piRNAs could be engineered to bind PIWI proteins to silence PIWI genes and block harmful outcomes. Secondly, at the transcriptional level, the PIWI/piRNA system might serve as a potential tool for future research to improve human health by manipulating DNA methylation of certain genes. We might be able to hypermethylate the promoter CpG island of PIWI to reduce its expression, thereby blocking piRNA and PIWI binding and its downstream steps. Unlike genetic alterations, DNA methylation is reversible, which makes it extremely interesting for therapeutic approaches. Last, at the posttranscriptional level, piRNAs could block the synthesis of cancer-related proteins through a mechanism that binds to mRNAs, similar to the mechanism by which miRNAs do not require Dicer. Although studies into piRNAs-based therapeutic methods are still in its early stages, more therapeutic outcomes could be achieved in the future as the mechanisms and functions of piRNAs in cancer are increasingly understood.

## Conclusion and perspective

Due to the advent of next-generation sequencing technologies, the expression of piRNAs can be easily observed. In fact, the study of piRNAs has deepened our understanding of the complex regulatory network of non-coding RNAs. Numerous studies have reported dysregulated expression of piRNAs in different diseases samples and proposed the underlying mechanisms. In this review, we summarized that piRNAs could guide PIWI proteins to regulate gene expression in a transcriptional way including DNA methylation and histone methylation (Fig. 2a, b) and/or posttranscriptional way including mRNA degradation and m6A methylation and/or posttranslational modifications (Fig. 2c, d). Under physiological conditions, the piRNA/PIWI complex formed by inherent piRNAs can establish an inhibitory chromatin state by histone modification to silence TEs and maintain genomic stability. When certain disease occurs, piRNAs expressions aberrantly increase but, in the others, piRNAs have been seen to be downregulated or they may be absent totally. Newly generated or intrinsic piRNA/PIWI complex affects the expression of different genes through global DNA hypomethylation, gene-specific DNA hypermethylation or posttranscriptional way and they play various roles in different pathophysiological processes, signaling pathways, the cell cycle, DNA repair, cell adhesion, invasion, and metastasis etc. Nevertheless, it is of note that translation and post-translational modification are equally important for the occurrence and development of tumors, so we need to conduct more in-depth research on the level of translation and post-translational modification of piRNAs in the future. piRNA/PIWI complex have a close influence on the occurrence of a variety of diseases including cancers. Owing to the heterogeneity of cancer, piRNAs can be both repressors and promoters in diverse cancers. Actually, the exact molecular mechanisms by which piRNAs are disregulated and impact on human health are still in its fancy and require further investigation. In addition to environmental and genetic factors, epigenetic changes also play a pivotal role in the occurrence of diseases, among which DNA methylation occupies a special position. We also summarized the role of piRNA/PIWI in the occurrence and development of cancer and other human diseases from the perspective of DNA methylation. In addition, there is growing evidence that the expression of piRNAs and methylation levels in some cancer-related genes are related to pathological variables or clinical outcomes; for that reason, they may have significant clinical value as diagnostic biomarkers. Moreover, piRNAs' regulation of disease through DNA methylation appears to offer new insights into epigenetics and great potential for future interventions in disease processes, including cancer.

However, the potential applications of piRNAs for clinical medicine still face plenty of challenges. First of all, it remains unclear that whether the abnormal expression of piRNAs really participate in the development of these diseases or just are a byproduct of other molecular activities. Secondly, to a large extent, the molecular mechanisms of physiological and pathological action of piRNAs are largely unknown, while some studies have proposed potential molecular mechanisms. Thirdly, in clinical practice, the potential application of piRNAs as a diagnostic and therapeutic target is currently at a speculative and theoretical stage, such as how to distinguish between healthy individuals and patients and how to resolve potential side effects, drug resistance, therapeutic effects, and administration mode. Accordingly, in the future, further studies and multi-center clinical trials should be conducted to fully aware of the regulatory mechanism and clinical application of piRNAs.

## Figures and Tables

**Table 1:** Dysregulated piRNAs in cancers and other diseases

piRNA	Disease	Dysregulation	Gene	Mechanism	Role	Reference
piR-823	Breast cancer	up	APC	Increases the expression of DNMTs, promotes DNA methylation of APC gene, thereby activates WNT signaling, induces CSCs and contributes to tumorigenesis	promotor	77
piR-932	Breast cancer	up	Latexin	Reduces the expression of Latexin through methylation its promoter region CpG island, induces CSCs and affects metastasis of breast cancer	promotor	78
piR-021285	Breast cancer	down	ARHGAP11A	Inhibits cell invasion and proliferation methylating by ARHGAP11A	suppressor	79
piR-sno75	Breast cancer	up	TRAIL	Upregulates TRAIL by inducing H3K4 methylation/H3K27 demethylation and inhibits tumor growth	suppressor	80
piR-34871, piR-52200, piR-35127, piR-46545	Lung cancer	up	GMIP	Regulates DNA methylation of oncogenes and anti-oncogenes, initiates local tumor growth and inhibits cell apoptosis	promotor	92
		down	A4GALT			
piR-823	Multiple myeloma	up	p16INK4A	Increases DNMT3A and 3B, promotes DNA methylation and increases the tumorigenesis	promotor	44, 102
Has_piR_011186	Leukemia	up	CDKN2B	Upregulates the methylation of DNA and histone H3 including H3K9 and H3K27 in the region of CDKN2B promoter	promotor	103
piR-823	Esophageal cancer	up	-	Induces aberrant DNA methylation through DNMT3B and plays tumor oncogenic role	promotor	45
piR-823	Gastric cancer	up	-	Causes cells aberrant “stem-like” state by reducing tumor supporter genes methylation and inhibits proliferation of cancer cells	suppressor	110, 111
piR-31470	Prostate cancer	up	GSTP1	Recruits DNMT1, DNMT3A and methylate CpG island of GSTP1	promotor	117
DQ589977, DQ591415, DQ598918, DQ601291, DQ601609	Testicular cancer	down	PIWIL1, PIWIL2, PIWIL4, TDRD1	Promoter CpG island hypermethylation-associated silencing	promotor	118
Aplysia piRNAs	Long-term synaptic facilitation	up	CREB2	Facilitates CpG island methylation in the CREB2 promoter	promotor	132
DQ541777	Neuropathic pain	up	CDK5rap1	Increases the methylation level of CpG islands by recruiting DNMT3A to CDK5rap1 promoter	promotor	134
piR-007899, piR-019162	Gulf War Illness	up	-	Alteration in DNA methylation and piRNAs could be detected	promotor	135
piR-63076	Pulmonary hypertension	up	Acadm	Attributes to Acadm DNA methylation and finally increases hypoxia-induced PASMC proliferation	promotor	139
td-piR(Glu)	Immune	up	CD1a	Recruits SETDB1, SUV39H1, and HP1b to the the region of CD1a promoter and facilitates H3K9 methylation	-	140

**Table 2:** Dysregulated PIWI in cancers and other diseases

PIWI	Disease	Expression	Gene	Function	Reference
PIWIL1/HIWI	IDCs	up	-	Promotes cancer development by aberrant DNA methylation	85
	Lung adenocarcinoma	up	-	PIWIL1 expresses aberrantly due to promoter DNA hypo-methylation and promotes proliferation, invasion and migration	95
	Colorectal cancer	up	-	PIWIL1 gene ectopically activates due to promoter demethylation and correlates with tumor differentiation, lymphnode invasion and metastasis; cancer-specific immunotherapies	108
	Endometrial cancer	up	PTEN	Causes PTEN underexpression through DNMT1-mediated hypermethylation	120
	NSCLC	up		Expression could be partly upregulated by methylation	96
PIWIL2/HILI	IBCs	down	-	Probably associated with down-regulation of DNMT1, histone H1, HP1 and SUV39H1	83
	IDCs	up	-	Aberrant DNA methylation	85
	Cervical cancer	up	c-Myc, Nanog, Oct4, Sox2, Klf4	Induces H3K9 acetylation but reduces H3K9 trimethylation	119
	Melanoma	up	MAGEA	Methylates LINE-1, to regulate MAGEA expression and inhibits melanoma cell migration	44
	Behavioral deficits	down	LINE-1	Hypomethylates intergenic areas and LINE-1 promoter areas	133
PIWIL4/HIWI2	IBCs	down	-	Probably solely associated with down-regulation of DNMT1,	83
	NSCLC	down	-	Positively correlated with overall methylation and poor outcomes	96

## Abbreviations

Ago3: Argonaute 3
Aub: Aubergine
CDK5rap1: CDK5 regulatory subunit-associated protein 1
CpG: 5'-cytosine-phosphate-guanine-3'
CSCs: Cancer stem cells
CVDs: Cardiovascular diseases
DNMT: DNA methyltransferase
EMT: Epithelial-mesenchymal transition
H3K9me3: Histone 3 trimethylated on lysine 9
LINE-1: Long interspersed nuclear elements-1
miRNAs: MicroRNAs
Mino: Minotaur
OS: Overall survival
Panx: Panoramix
piRNAs: Piwi-interacting RNAs
PTEN: Phosphatase and tensin homolog deleted on chromosome ten
PTGS: Post-transcriptional gene silencing
PTMs: Posttranslational modifications
RISC: RNA-induced silence compounds
ncRNAs: Non-coding RNAs
siRNAs: Small interfering RNA
TEs: Transposable elements
TGS: Transcriptional gene silencing
Zuc: Zucchini
